# New Design of a Soft Robotics Wearable Elbow Exoskeleton Based on Shape Memory Alloy Wire Actuators

**DOI:** 10.1155/2017/1605101

**Published:** 2017-09-05

**Authors:** Dorin Copaci, Enrique Cano, Luis Moreno, Dolores Blanco

**Affiliations:** Department of Systems Engineering and Automation, Carlos III University of Madrid, Madrid, Spain

## Abstract

The elbow joint is a complex articulation composed of the humeroulnar and humeroradial joints (for flexion-extension movement) and the proximal radioulnar articulation (for pronation-supination movement). During the flexion-extension movement of the elbow joint, the rotation center changes and this articulation cannot be truly represented as a simple hinge joint. The main goal of this project is to design and assemble a medical rehabilitation exoskeleton for the elbow with one degree of freedom for flexion-extension, using the rotation center for proper patient elbow joint articulation. Compared with the current solutions, which align the exoskeleton axis with the elbow axis, this offers an ergonomic physical human-robot interface with a comfortable interaction. The exoskeleton is actuated with shape memory alloy wire-based actuators having minimum rigid parts, for guiding the actuators. Thanks to this unusual actuation system, the proposed exoskeleton is lightweight and has low noise in operation with a simple design 3D-printed structure. Using this exoskeleton, these advantages will improve the medical rehabilitation process of patients that suffered stroke and will influence how their lifestyle will change to recover from these diseases and improve their ability with activities of daily living, thanks to brain plasticity. The exoskeleton can also be used to evaluate the real status of a patient, with stroke and even spinal cord injury, thanks to an elbow movement analysis.

## 1. Introduction

The interest in developing new robotic devices for medical rehabilitation is due to their capacity to perform repetitive tasks and because they allow to analyse the patient evolution objectively [1]. The most outstanding medical applications for an exoskeleton are as follows:

- Stroke rehabilitation (ischemic and hemorrhagic ictus), thanks to brain plasticity to regenerate neurons in an anatomic and functional way. It is the most interesting application, and it is analysed particularly in the following sections
- Muscle injury recuperation
- Spinal cord injury recovery
- Muscular stimulation for aged people

Stroke has become one of the most common diseases and is a cerebrovascular accident (CVA) affecting lots of victims, not only with deaths but even disabling people, in an increasingly aged society. WHO (World Health Organization) calculates that 15 million people have stroke every year in the world. Stroke happens when poor blood flows to the brain resulting in cell death. There are two main types of stroke: ischemic, due to the lack of blood flow, and hemorrhagic, due to bleeding. Signs and symptoms of stroke may include an inability to move or feel on one side of the body, problems in understanding or speaking, feeling like the world is spinning, or loss of vision to one side, among others. The long-term effects of stroke are loss of memory and up to 85% motoric disturbances on the opposite side of the body to the area of the brain where there was a stroke [2]. This disorder is called hemiparesis or hemiplegia, which is a unilateral paresis and weakness of the entire left or right side of the body and it is eventually characterized by uncontrolled or partial movements. The weeks following a stroke are crucial because the damage can be evaluated. Motoric disturbances are divided into weakness, loss of joint control, and uncontrolled muscle contractions.

The disabilities are caused by weakness and abnormal muscle contractions, due to the production of interruptions in the upper limb movements and loss of dexterity of the hand [3].

The individual movement capacity is essential to perform ADL (activities of daily living). Motoric disturbances produced by stroke decrease significantly the quality of life of patients. These disorders cause difficulties in performing their daily activities. In this way, orthoses and exoskeletons, along with physical rehabilitation and muscle electrical stimulation techniques, are currently being developed in order to improve the physical condition of patients and to improve their quality of life consequently. It has been demonstrated that with physical rehabilitation, either carried out by using exoskeletons or wearable devices or by the help of a therapist, stroke patients can recover their motoric capacity.

Therefore, these rehabilitation devices can improve the motoric condition of stroke patients thanks to brain plasticity, which is the nerve cell's capacity to automatically regenerate in an anatomical and functional way as a consequence of environmental stimulations (in this project, the stimulation is produced by the movement of the right arm by using the SMA exoskeleton). Neurons from a nondamaged brain area can develop the abilities of neurons from a damaged brain area by stimulating the muscles of the affected extremity [4–6].

In the last decade, some devices were developed for elbow joint medical evaluation and rehabilitation therapy. A broad review can be found at [1, 7]. Most of them are like stationary systems (e.g., Armeo Power from Hocoma (https://www.hocoma.com/solutions/armeo-power/) and MEDARM [8]), and other parts are like portable devices (e.g., NEUROExos [2]). In terms of mechanics, the most common ones are the end-effector devices, which connect the subject's limb with the rehabilitation device at its most distal part (e.g., MIT Manus [9] and ACRE [10]), and the exoskeleton-based devices where the skeletal structure of the limb coincides with the mechanical structure of the rehabilitation device [2]. The actuation system of this type of devices is mainly based on electrical motors but also on devices which can be found to be actuated with hydraulic actuators and pneumatic actuators, where a special part is represented by the pneumatic artificial muscle (PMA) and series elastic actuator (SEA). In addition, the functional electrical stimulation (FES) is also used, but, with this technique, it is difficult to achieve precise and repeatable movement and it may be painful for the patient [1].

**Figure 1:**
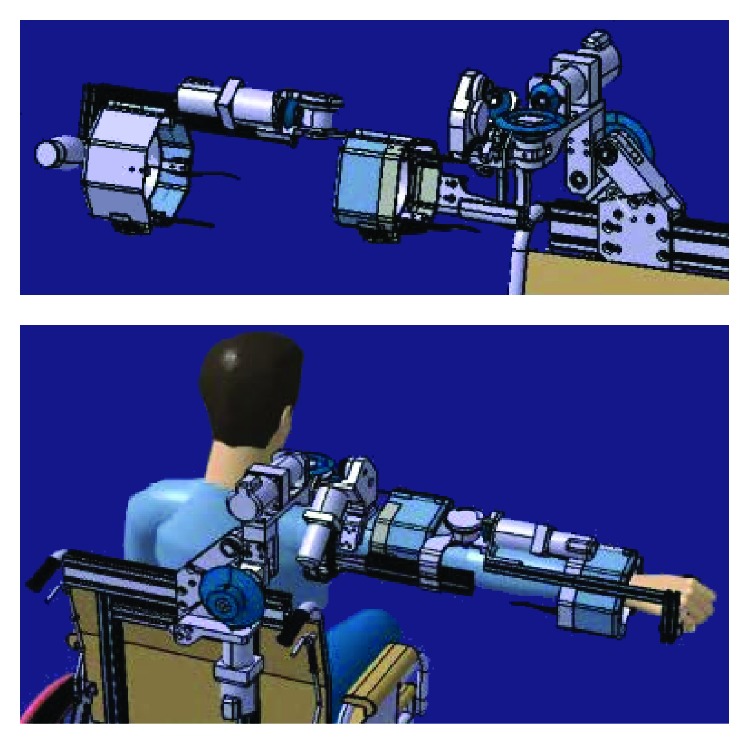
Exoskeleton design developed by Moubarak et al. [11].

In Figure 1, an example of a current exoskeleton for medical rehabilitation of an upper limb extremity (elbow and shoulder: 4 DOFs), which is characterized by a weight of 10 kg, is shown; it is attached to a wheelchair and it is actuated by brushless DC motors with gears.

In case of [12], this 9-DOF exoskeleton for medical rehabilitation of an upper limb extremity (shoulder, elbow, and wrist) with a weight of 23 kg is attached to the wall, and it is actuated by DC motors with gears. It is sensorized with EMG sensors. As the same way, EXO-UL7 [13] is a 7-DOF exoskeleton for the shoulder, elbow, and wrist simultaneous rehabilitation, and it is actuated by DC motors with gears. Garrec et al. [14], Gupta and O'Malley [15], and Perry and Rosen's [16] studies are similar examples to the previous ones. On the other hand, the 4-DOF NEUROExos [2] is actuated by hydraulic cylinders and the 5-DOF RUPERT IV [17] is actuated by PMA actuators.

To ensure elbow joint alignment axes with the exoskeleton axes, solutions such 4-degrees-of-freedom passive mechanism [2] were proposed. This solution is a rigid structure which implicates a complex mechanism with various mechanical articulations and elevated weight.

These exoskeletons are characterized by too much weight, nonportability, and noncomfortability, because of their weight, number, and noise of actuators (mainly in the case of DC motors). This shows that new types of actuators are required in order to improve comfortability and to reduce weight. SMA-actuated exoskeleton can be seen as a possible solution for these drawbacks [18].

In order to avoid all of those problems (weight, cost, portability, and comfort), the objective of this project is to design and build a low-cost elbow medical rehabilitation exoskeleton with one degree of freedom for flexion-extension movement actuated with shape memory alloy wires. This device is based on soft robotics, which is a subfield of robotics that researches new types of actuators based on deformations of materials, and it is wearable, light, and noiseless.

The proposed exoskeleton in this paper represents an alternative solution for the exoskeleton presented in the research in [19]. This new solution is inspired by the concept defined by Ammar: “SAS: SMA aiding sleeve” [18]. The developed exoskeleton is formed by a 3D-printed structure which is set directly on the arm, and it can be adapted thanks to tip-top buckles. The operating principle of SMA wire actuators is a SMA material that can vary its internal structure because of a phase change, which, in this case, is produced by a temperature increase based on the Joule effect. This phase change produces a contraction of SMA wire length. When the SMA wires contract, the arm movement is produced directly. Pulleys are not required. The arm is protected from heat by a sleeve.

A shape memory alloy (SMA) is an alloy that remembers its original shape and that when deformed returns to its predeformed shape when heated above its transformation temperature, due to a transition between a martensite phase (at low temperature) and an austenite phase (at high temperature). The most commonly used SMA alloy for actuation is Nitinol, a result of a nickel and titanium alloy. The deformation-recovery cycle of Nitinol can be repeated millions of times, whether the applied deformations are in the recovery range of the SMA, thanks to its superelasticity [20].

A SMA actuator uses a SMA element (wire or spring) as the transducing material of the actuator—the SMA transducer converts thermal energy into mechanical work. The SMA is heated when electric current is flowing through the SMA (Joule effect). Then, two transduction processes take place. First, electric energy is transformed into thermal energy thanks to the Joule effect. This thermal energy triggers the shape recovery process of the SMA element, and the resulting recovery energy is transformed into mechanical work [21].

Using this type of actuation, we solve some of the actual limitations shown by commercial devices, such as the total weight of the device, the operation noise, ergonomic physical human-robot interface, and the financial costs. These points can be viewed as important contributions of this work. An additional advantage is that the proposed system has a simple structure with an easy track for setup, considering that the setup time needs to be less than 5 minutes.

In this paper, first of all, the design of the elbow exoskeleton actuated with SMA wires is presented, including the elbow biomechanic simulation, SMA actuator operation, and sizing. Then, the control strategy is developed, including the description of the hardware (control electronics, power electronics, and feedback sensor) and the control scheme. Finally, the preliminary results of the experimental tests are analysed.

## 2. Methodology

In this section, the most important items of the exoskeleton design will be explained. The biomechanic simulation to estimate the necessary torque to provide the expected range of movement, the SMA-based actuator design and sizing, and the crimping process are also discussed.

### 2.1. Biomechanics

Nowadays, in the development of any robotic device, simulation tools play an important role due to their capacity to analyse the expected performance of the system designed prior to manufacturing. To estimate the necessary torques in the articulations for a specific patient, a simulation software biomechanics of bodies (BoB) was used [22]. BoB is the human musculoskeletal modelling in MATLAB/Simulink. BoB is formed by 36 links of the skeleton and 666 muscles. This tool has been developed by Coventry University (UK), and it has two versions: one for inverse dynamics and another for direct dynamics. In the inverse dynamic model, BoB calculates the torque of articulations, muscular load distribution, and contact forces in joints. The software is capable of simulating the inverse dynamic behaviour of the human body, receiving as input the height, weight, and motion of the patient and giving as output, among other data, the torque of articulations. In the forward dynamic model, BoB can calculate the movement which is caused by the muscular activation and the external force action. As a result, BoB is a very powerful tool, and it has a very potent graphical capability and data postprocessing [23].

**Figure 2:**
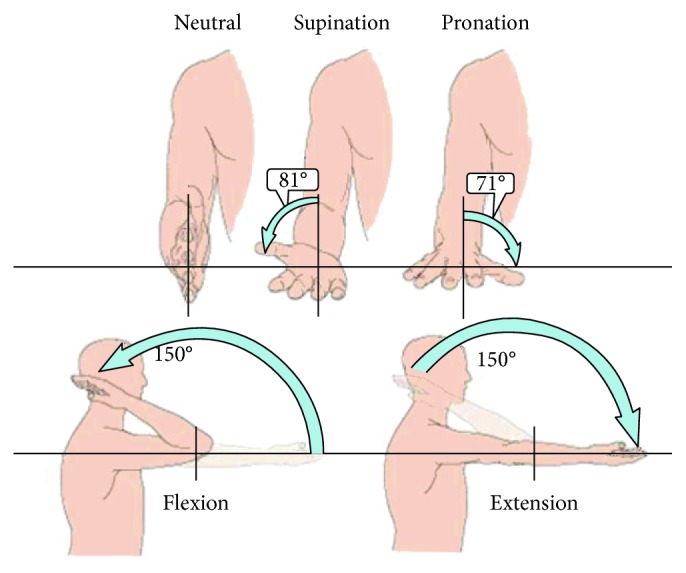
Normal range of motion for the elbow joint.

In particular, elbow articulation is the joint between the humerus in the upper arm and the radius and ulna in the forearm which allows the hand to be moved towards and away from the body. The elbow joint is characterized by 2-DOF; on the one hand, one degree of freedom for flexion-extension movement and, on the other hand, one degree of freedom for pronation-supination movement [24]. The angular range for flexion-extension movement is estimated to be between 0 and 150 degrees, but in the ADL, the functional range is estimated to be between 30 and 120 degrees. In the pronation-supination movement, the averages are 71 degrees for pronation and 81 degrees for supination (Figure 2). Usually in ADL, the total range of pronation is approximately 50 degrees and for supination, 50 degrees [25].

In this case, the simulation was configured with the following parameters: weight 80 kg, height 1.8 m, and a trajectory in the right elbow joint between 0 and 150 degrees with a frequency movement of 0.25 Hz. In addition, a force of 20 N was applied.

**Figure 3:**
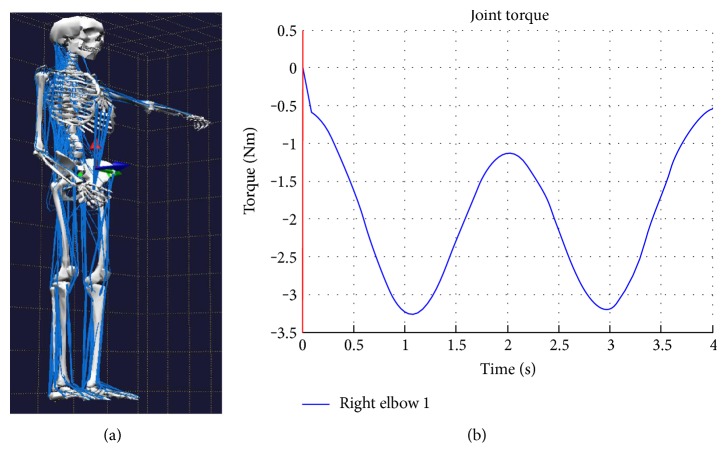
(a) BoB simulator configured in the flexion-extension of the elbow joint. (b) Simulation results with the necessary torque in the elbow joint.

As seen from the simulation, to complete the rehabilitation task in the elbow articulation successfully, a torque of approximately 3.5 Nm is necessary (Figure 3). This case assumes that the patient has definitively lost the motor function, and all the forces are made by the exoskeleton.

### 2.2. SMA Actuator

The proposal for an exoskeleton that uses a nonconventional actuation system based on SMA wires requires an analysis of the advantages and disadvantages compared to other more conventional actuators. In addition, it is necessary to design the actuator itself according to the specific requirements of the device.

#### 2.2.1. Advantages and Disadvantages

The main advantages of a SMA actuator [26] against a DC motor are as follows:

- Lower weight: as SMA actuators are built with SMA wires, they are lighter and any other elements like gears are not necessary.
- SMA actuators are characterized by a reduction in size, weight, and complexity of the robotic devices.
- Low noise level: their silent performance and being lightweight are the most important advantages in building an exoskeleton for medical rehabilitation.
- The torque-weight relation for a SMA actuator is excellent.
- SMA wires can absorb unexpected axial efforts.
- SMA exoskeleton cost is relatively cheap.
- Power electronics and control system are easy.

On the other hand, the problems of SMA actuators are as follows [26]:

- SMA materials have nonlinear behaviour due to the hysteresis phenomena that happen in heating/cooling processes in martensite-austenite phase changes. This is the main reason why the control of these actuators is more difficult than that of DC motors.
- Control depends on temperature (room temperature) in heating/cooling processes, so it is necessary to perform an analysis to optimize the heating/cooling speed and the absorption-dissipation energy.
- Low actuation frequency and limited bandwidth. It is influenced by SMA heating/cooling speed.
- Energy is inefficient.
- Ideal physical disposition design for the SMA actuator is indispensable, and it is not an easy process.

#### 2.2.2. SMA Actuator Design

**Figure 4:**
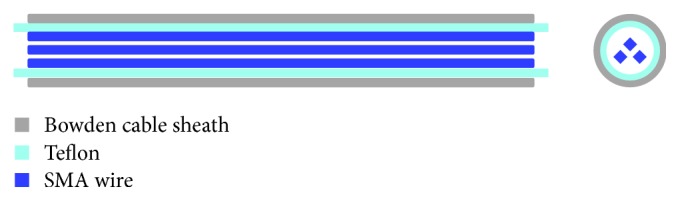
SMA actuator elements.

The actuator based on SMA wires used in this work is formed by the following elements (see Figure 4()) [21]:

- Bowden cable: a Bowden cable is a mechanical transmission system which consists of an internal flexible metal and an external flexible plastic cover. The main function of a Bowden cable is to guide the SMA wires to ease the mechanical transmission in case of contraction to generate the movement for the actuator and to decrease the mechanical losses which are produced by the reduction of the SMA wire tension [21].
- Teflon sheath: the Teflon sheath allows isolating the SMA wires electrically from the Bowden cable (the SMA wires are connected to the power supply which, with the aid of the Joule effect, increases their temperature). Moreover, Teflon is characterized by its high temperature resistance capacity (up to 260°C) without suffering plastic deformation due to the heat effect. It also has a very low coefficient of friction, so the movement transmission is going to be easier for a SMA wire when it contracts [27].
- SMA wires: there are different types of SMA actuators depending on the temperature of the wire (high and low temperatures), number of wires, different thicknesses or internal diameter, and so forth, in order to optimize the weight, energy consumption, or torque generated by the actuator.

### 2.3. Exoskeleton Design

The steps to design and build a SMA-based exoskeleton are as follows:

- SMA actuator sizing should be done: length, electrical power supply, electric resistance, and number of wires.
- Design the structure based on anthropomorphic measures while taking into account the comfortability for the medical rehabilitation process. The sensorization of the elbow joint is also carried out.
- Adjust the control system in the experimental tests.

#### 2.3.1. SMA Actuator Sizing

In order to characterize the type of the SMA actuator, it is necessary to take into account that a human arm represents 5.7% of the total body weight in men and 4.97% in women [28]. In this case, our trials are carried out over a male subject which has a 80 kg total weight and whose right arm weighs approximately 4.5 kg. A 0.02-inch diameter, activated in high temperature (90°C), SMA wire has a pull force of 3.56 kg (34.9 N) [29]; then, if a SMA actuator is built with *n* = 3 wires, it can perform the flexion-extension movement for the elbow joint, and it has an acceptance range for a wide group of patients, depending on their age, gender, height, and weight.

In order to calculate the SMA wire length for the actuator, the exoskeleton must be worn by the trial and the structure should be set to the correct position for the biceps and forearm. In the case of the selected subject, the contraction distance needed for a flexion-extension movement for the elbow joint, considering a range of movement from 0 to 150 degrees, is around 7.5 cm, so *C* ≈ 7.5 cm.

**Figure 5:**
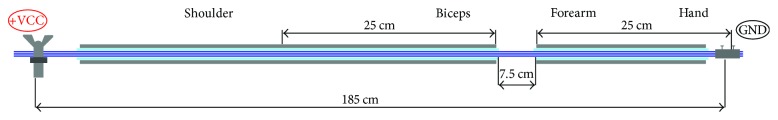
SMA actuator.

Next, it is necessary to calculate the SMA wire length (*L*). For that, it is important to know that a SMA wire can vary until 4% of its total length [29].  Figure 5 presents the actuator with its dimensions.

(1) $$\begin{array}{c} L=\frac{C}{4/100}\approx 25\cdot C\approx 25\cdot 7.5\;cm\approx 185\;cm\approx 1.85\;m. \end{array}$$

#### 2.3.2. Design the Exoskeleton Structure

**Figure 6:**
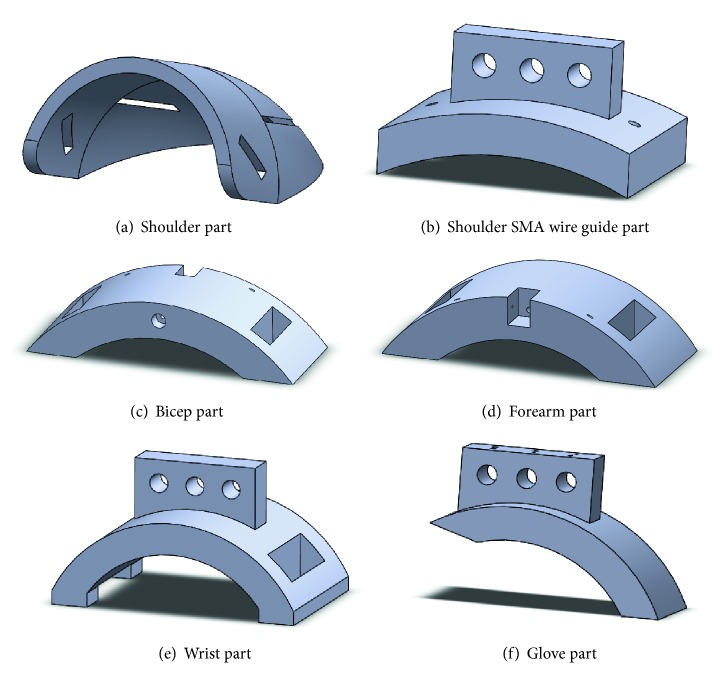
Elbow exoskeleton 3D-printed parts.

The exoskeleton is built with parts which are presented in Figure 6.

**Figure 7:**
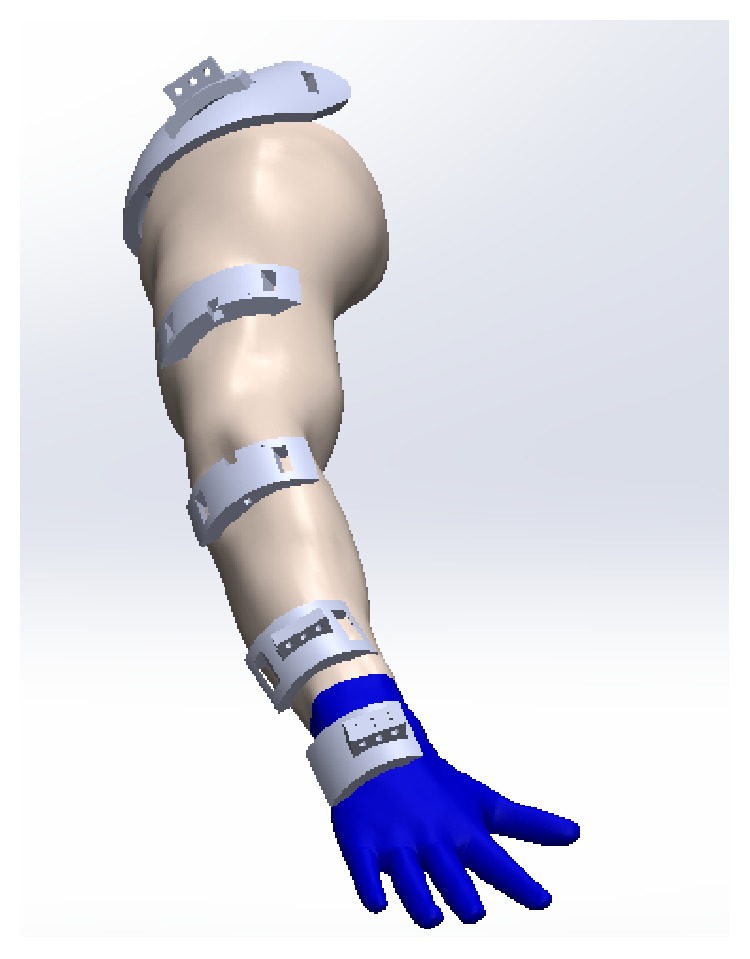
Design of a wearable exoskeleton for elbow medical rehabilitation.

**Figure 8:**
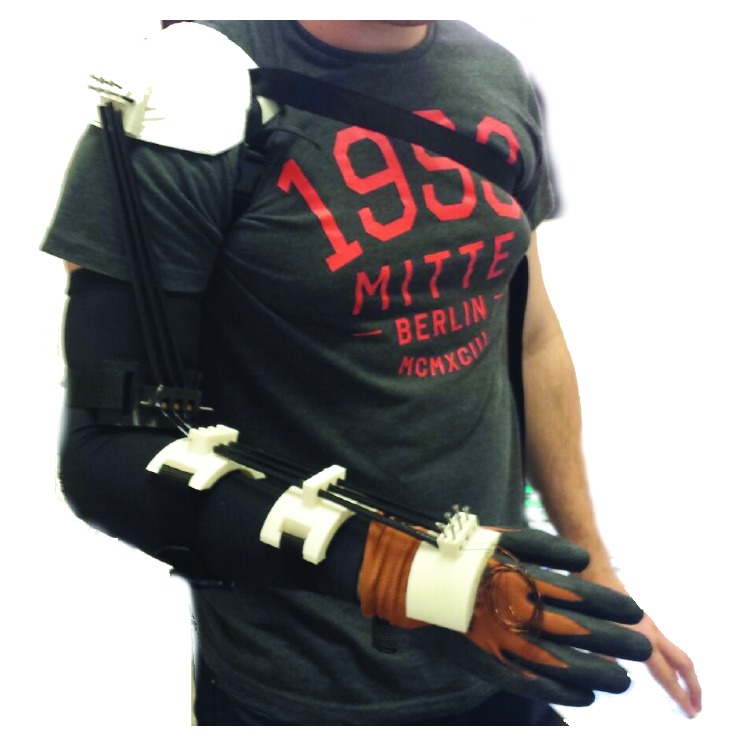
Wearable exoskeleton for elbow medical rehabilitation with shape memory alloy actuators.

The main function of the shoulder part defined in Figure 6(a) is to fix the exoskeleton to the patient's arm, in order to avoid the exoskeleton parts' displacements and to optimize the movement produced by the SMA wires' contraction. The part defined in Figure 6(b) is placed over the shoulder part as shown in Figure 7, and its main utility is to guide the SMA actuators and to minimize mechanical losses. Parts defined in Figures 6(c) and 6(d) are used to fix the exoskeleton to the human body and to ease the movement generation of the SMA actuators while memory effect is activated. Next, the part defined in Figure 6(e) is essential. As the exoskeleton is built for flexion-extension elbow medical rehabilitation, the degree of freedom for supination-pronation must be cancelled, so the patient's wrist is blocked. Finally, the part defined in Figure 6(f) is fixed to a glove over the hand and it is used for the crimping process. Every part must be placed according to Figure 7, and the real built exoskeleton is shown in Figure 8.

The estimated weight for this exoskeleton is approximately 0.6 kg, without taking into account the electronics. This low weight eases the comfortability and medical rehabilitation process for the patients.

Crimping process: the crimp process is the technique which allows to keep tight the SMA wires so that, when the wires contract, there are no mechanical losses and that contraction is exclusively used to generate the actuator movement.

**Figure 9:**
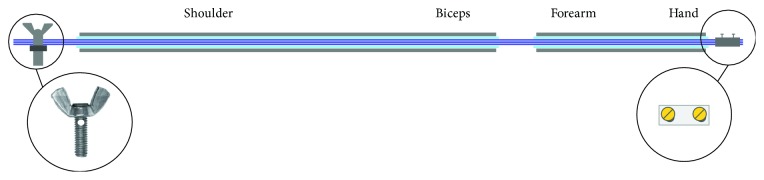
SMA wire crimping process for elbow exoskeleton actuators.

In this project, the crimping system for SMA wire-based actuators for an elbow medical rehabilitation exoskeleton is built with two parts: for crimping the SMA wires on the hand glove part, a metallic piece of an electric connector, which is put inside the part defined in Figure 6(f), is being tightened with the wires with two screws. For crimping the SMA wires on the shoulder part, a four-metric thumbscrew is necessary, as shown in Figure 9.

The crimping process is as follows:

- First, the exoskeleton is placed over the patient's damaged arm and the end of the SMA actuator is fixed to the glove over the metallic piece, previously described.
- Next, the patient moves his arm up to the initial position with extended elbow (*θ*_(*k*)_ = 0 degrees) and the SMA wires are tensioned without tightening the screw.
- Finally, the thumbscrew and the nuts are tighten, and now, the SMA wires are totally tensioned and the patient arm is at the initial position.

Finally, the experimental tests for the control system adjustment are presented in Section 3.

## 3. Control Strategy

In this section, the description of the hardware system is detailed: control electronics, power electronics, and feedback loop sensor. The exoskeleton system is also analysed from control engineering's point of view.

### 3.1. Hardware

#### 3.1.1. Control Electronics: STM32F4DISCOVERY

STM32 family, which is formed by 32-bit flash microcontrollers with Arm Cortex-M architecture, has been designed to offer new degrees of freedom for DSC (digital signal controllers) microcontroller users. It is characterized as having high efficiency, real-time capacities, signal digital processing, low power consumption, low voltage operation, with a completed integrity, and easy development [30].

The control system, previously designed, has been implemented thanks to a methodology based on hardware and software tools of rapid control prototyping (RCP). This control system has been developed by Carlos III University of Madrid [31], and it is based on a very powerful and affordable 32-bit microcontroller: STM32F4, by STMicroelectronics, previously presented. The embedded system is responsible for total control, data acquisition, and processing for the designed actuators. Usually, a RCP system provides a higher level of abstraction than other methods thanks to the usage of graphical programming languages. In this case, the graphical programming language to develop an embedded controller is MATLAB/Simulink. Thanks to UC3M-RCP, the code can be generated, compiled, and loaded on the microcontroller in a completely automatic way for the user.

The SMA materials are nonlinear and are characterized by the hysteresis phenomena in heating/cooling processes in martensite-austenite phase change leading to a complex control system. The control is carried out by PWM (pulse-width modulation), which is a modification of the on/off control, by defining a frequency for the control signal. In this way, by varying the frequency and pulse width of the control signal, the system (exoskeleton) can be controlled. In this project, the control signal is generated by the BPID controller (*bilinear proportional-integral-derivative controller*), which was used successfully in the control of SMA [32].

All the necessary code: the control algorithm, the position sensor, and the bidirectional communication between the STM32F4DISCOVERY and the PC were implemented with the aid of UC3M-RCP. The control algorithm receives the desired angular position reference of the exoskeleton (which coincides with the elbow joint) and permits data acquisition of the angular position and velocity of the elbow joint. This data is stored and used in the patient evaluation analysis.

#### 3.1.2. Power Electronics

The most common method to activate the SMA wire and its shape memory effect consists of heating them with the Joule effect. Here, two transduction processes take place. First, electric energy is transformed into thermal energy thanks to the Joule effect. After that, the thermal energy is transformed into mechanical work. In function of the diameter and the alloy type, the actuator can exert different forces. A power electronic PCB is used to generate the electrical current that it is going to flow through the SMA wires. It is formed by 16 PWM channels. Every channel is formed by a MOSFET transistor (STMicroelectronics STP310N10F7) which works as a commutation circuit and amplifies the PWM signal generated by the controller [31].

#### 3.1.3. Flex Sensor

A flex sensor, by Spectra Symbol, is a resistive sensor whose resistance varies depending on the bending angle. The flex sensor resistance changes when the metal pads are on the external bottom of the curve. It is used in robotics because it light in weight and its simplicity. It is recommended to pin up the base to avoid damages on the connector due to flexion efforts.

In this project, the flex sensor is used to measure the exoskeleton angular motion which coincides with the patient elbow joint angle, for flexion-extension movements in real time. The sensor is positioned over the elbow joint with the aid of the arm covering. The sensor signal is used as a feedback signal for the control loop and, along with the reference signal, it is possible to calculate the error signal for every instance.

**Figure 10:**
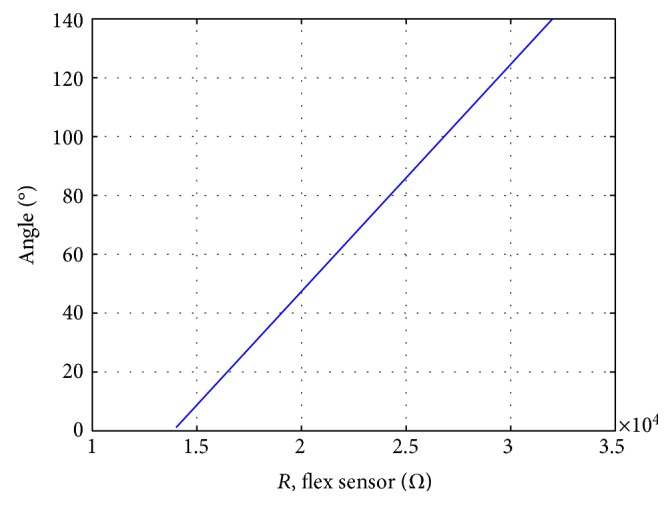
Flex sensor resistance depending on elbow angle.

The relationship between the elbow flexion angle and the electrical resistance is shown in Figure 10.

**Figure 11:**
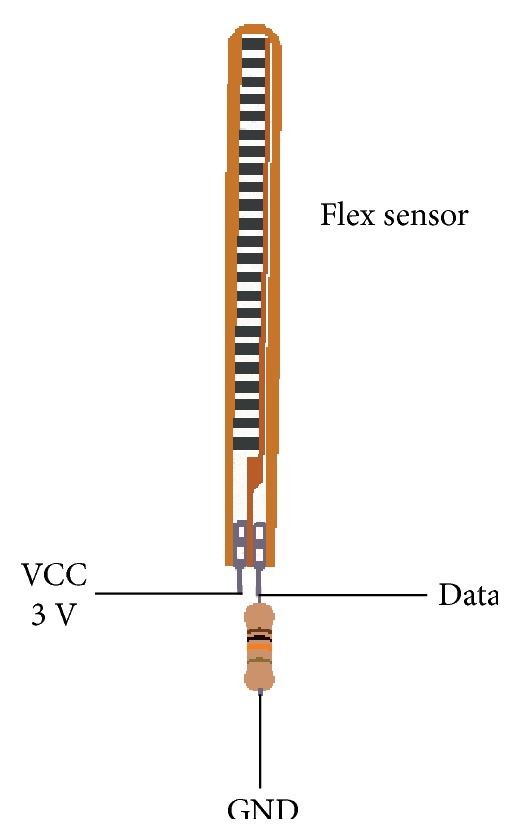
Electrical connections for the flex sensor.

Then, in Figure 11, the electrical installation for the flexion sensor with voltage divider configuration with a resistance *R* = 10 k*Ω*, with a 3 V power supply voltage, is shown. The power supply voltage is 3 V because the analog inputs of STM32F4 can only read signals with amplitudes of up to 3 V.

**Figure 12:**
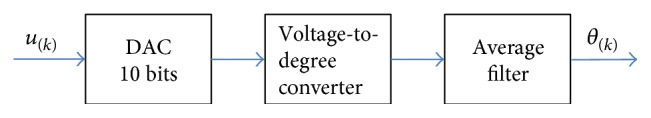
Data read sensor program for the elbow.

Thanks to RCP toolbox, the signal processing for the flex sensor is programmed with blocks in MATLAB as it is defined in Figure 12, where *u*_(*k*)_ is the voltage signal received from the flex sensor and the *θ*_(*k*)_ is the real angular position of the elbow. As it is shown in Figure 12, the electric signal from the flex sensor, which is read by STMicroelectronics board, is transformed into continuous values by a 10-bit digital-analog converter. Next, a conversion from voltage to degrees is necessary, taking into account the minimum and maximum resistance of the flex sensor, input voltage, and elbow range of movement. As it is a resistive sensor, the voltage variation of the sensor depending on the elbow position is linear. Finally, an average filter of 11 samples is applied to avoid peaks and to smooth the elbow signal position, although it produced a 0.11-second delay, taking into account that the sample time is *T*_s_ = 0.01 s.

**Figure 13:**
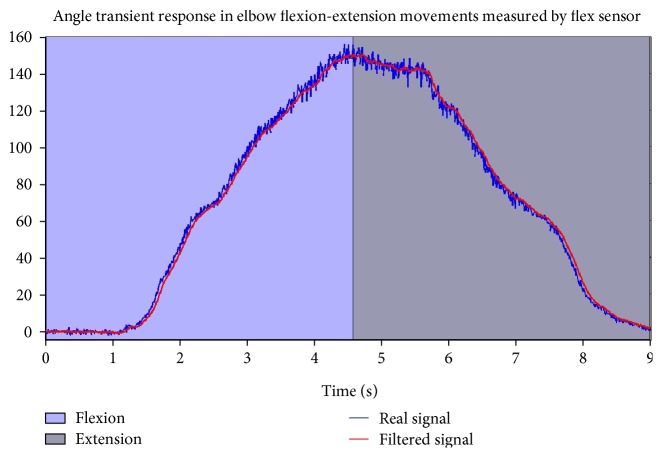
Elbow joint time response for flexion-extension movements.

Finally, the experimental response of the flex sensor to measure the elbow angle in flexion-extension movements is defined in Figure 13.

### 3.2. Control Scheme

Due to the large hysteresis area and heavily nonlinear behaviour of SMA actuators, obtaining an accurate mathematical model of the SMA actuator is a difficult task. In this paper, a type of bilinear controller consisting of a conventional PID controller cascaded with a bilinear compensator, known as BPID (bilinear proportional-integral-derivative controller), is used. Thereby, a SMA actuator, which is nonlinear, can be controlled by linearizing the plant. The BPID controller is simpler and easier to implement than other nonlinear control strategies [33].

**Figure 14:**
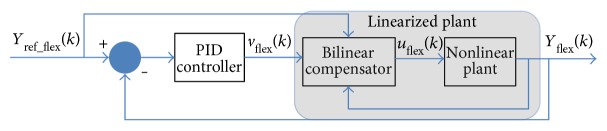
Control scheme for SMA-based wearable elbow exoskeleton (adapted from [33]).

The implementation of a BPID control system for an elbow exoskeleton with SMA wire actuators is shown in Figure 14, developed in MATLAB. Also, this control scheme has been successfully used in [32].

Every variable is defined as follows:

- *Y*_ref−flex_(*k*) is the reference signal set-point for the elbow position.
- *Y*_flex_(*k*) is the real elbow position measured signal by the flex sensor, and it is used in the control loop.
- *v*_flex_(*k*) is the linear PID control signal.
- *u*_flex_(*k*) is the PWM (pulse-width modulation) control signal that controls the system (exoskeleton).
- The nonlinear plant is the system formed by the exoskeleton 3D-printed structure and the SMA wire actuators.

## 4. Preliminary Results

The main objective is to build a SMA wire-based actuator exoskeleton for flexion-extension elbow medical rehabilitation, which is characterized by low weight (nearly 0.6 kg) and low noise level in operation in order to get comfortability, portability, and adaptability with low cost and innovative materials.

In this way, experimental tests are defined to check those characteristics of comfort and efficiency for a medical rehabilitation device.

**Figure 15:**
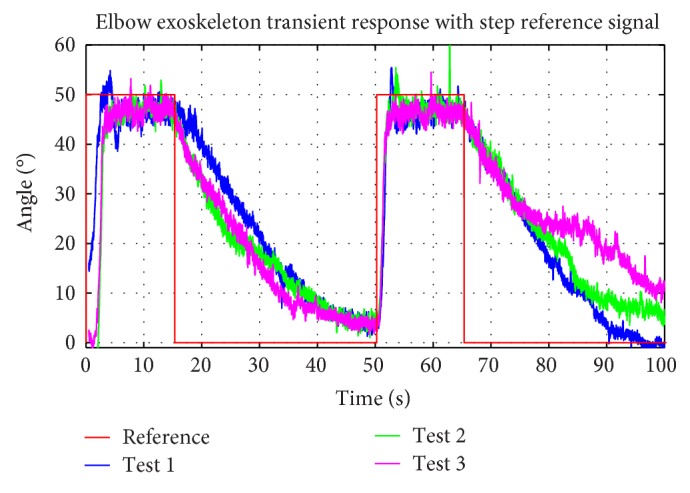
Elbow exoskeleton time response in experimental tests with step reference.

The preliminary results of the experimental tests carried out by a laboratory staff are shown below in Figure 15. The person characteristics are the following: 1.8 m height, male, with a weight of 80 kg, and 23 years old. A step reference signal is the input for the system and the response is analysed.

The reference signal is defined as a step signal due to the performance of the control system that can be easily analysed. If the SMA actuators respond fast and accurate to a step signal, the transient behaviour is expected to be controlled for heating/cooling processes for slower changes in the reference signal.

The elbow range of motion is approximately 150 degrees, and the SMA actuators are sized for this range. In this case, the reference signal set-point is 50 degrees. The difference between the reference signal set-point and the elbow range of motion is shown in this test. In the case of the CVA patients, usually the range of motion is limited and the recuperation of motor function is done by gradually increasing (step by step) the reference. For this reason, 50 degrees of reference represents a possible start in the process of rehabilitation therapy.

A comparative study of elbow exoskeleton time response for the step reference signal is carried out for three experimental tests in Figure 15. Result repeatability for SMA wire heating/cooling processes is observed. On the one hand, the heating process is defined as a 4.5-second time response due to the control system and power electronic actions on the SMA actuators. On the other hand, the cooling process is slower, almost 25 seconds. In this case, the cooling method is air convection at room temperature. Depending on the final application, there are several solutions to improve and accelerate the cooling process: the use antagonist actuator [34], that is, a double actuator formed by an actuator responsible for flexion movement and an actuator responsible for extension movement, taking into account the heating/cooling processes for each one to avoid wire breakages due to increasing stress when the wire is heated, or the use of springs to accelerate the recovery process of the original shape of the SMA wires. The average error in stationary state is 3.42 degrees in the case of positive steps.

**Figure 16:**
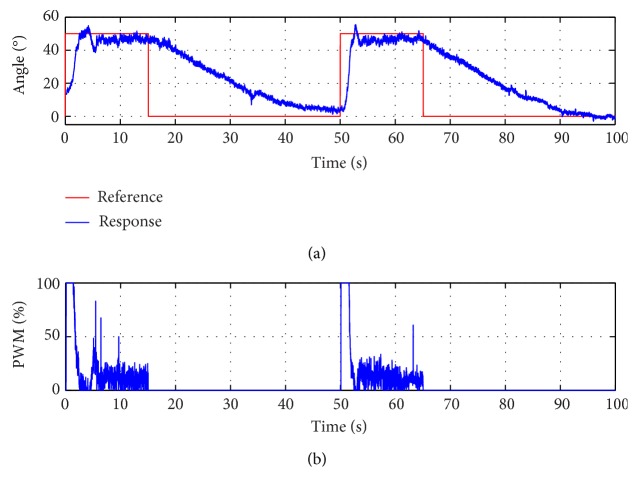
(a) Elbow exoskeleton transient response with step reference signal. (b) PWM control signal.

At last, the control signal is analysed for a step signal reference test (Figure 16), and it is observed that the PWM control signal is at maximum when it produced a step in the reference signal. In this instance, the power consumption of a SMA actuator is at maximum (160 W approximately, depending on the SMA actuators' voltage power supply). The power consumption is at minimum when the exoskeleton keeps the current position in a stationary state and during the cooling process.

## 5. Conclusions

In this paper, the preliminary design for a wearable elbow exoskeleton actuated with SMA-based actuators (without motors) which allows for the drastic reduction of the weight of the exoskeleton (about 0.6 kg) and achieving a quiet operation characteristic that increases the comfort and portability of the system was presented. In comparison with DC motors, SMA actuators are mainly lighter and more noiseless.

The proposed design of the exoskeleton does not use rigid components for the articulation motion, using the rotation center for proper patient elbow joint articulation. This has an important impact on the comfort of the human-robot interaction.

These reasons can be enough for their use in exoskeletons in order to favour the medical rehabilitation process for CVA patients by improving the comfortability and portability of these devices. Furthermore, the devices are cheap and can be built for each patient in less than one day with a 3D printer. The preliminary design of the exoskeleton was built as a low-cost medical system and easy-to-use rehabilitation device, with low-cost electronics and actuators, which can be adjusted depending on the patients.

Finally, the preliminary results show that this type of actuator can provide a suitable task performance for medical rehabilitation, though there are several pending problems (e.g., cooling process slowness) to build a completely functional exoskeleton. The results shown in this paper demonstrate that the proposed solution is viable.
